# Novel Variants of Clade 2.3.4 Highly Pathogenic Avian Influenza A(H5N1) Viruses, China

**DOI:** 10.3201/eid1912.130340

**Published:** 2013-12

**Authors:** Min Gu, Guo Zhao, Kunkun Zhao, Lei Zhong, Junqing Huang, Hongquan Wan, Xiaoquan Wang, Wenbo Liu, Huimou Liu, Daxin Peng, Xiufan Liu

**Affiliations:** College of Veterinary Medicine, Yangzhou University, Yangzhou, China (M. Gu, G. Zhao, K. Zhao, L. Zhong, J. Huang, H. Wan, X. Wang, W. Liu, H. Liu, D. Peng, X. Liu);; Jiangsu Co-innovation Center for Prevention and Control of Important Animal Infectious Diseases and Zoonoses, Yangzhou (M. Gu, X. Wang, W. Liu, H. Liu, D. Peng, X. Liu)

**Keywords:** influenza, avian influenza virus, clade 2.3.4, China, clade, influenza virus, flu, H5, H5N1, highly pathogenic, viruses, avian, AIV, phylogeny, chickens, birds, HPAI

## Abstract

We characterized 7 highly pathogenic avian influenza A(H5N1) viruses isolated from poultry in China during 2009–2012 and found that they belong to clade 2.3.4 but do not fit within the 3 defined subclades. Antigenic drift in subtype H5N1 variants may reduce the efficacy of vaccines designed to control these viruses in poultry.

Infection with the Asian lineage of highly pathogenic avian influenza (HPAI) A(H5N1) virus (prototype strain A/goose/Guangdong/1/96 [Gs/GD]) has resulted in substantial losses in the poultry industry and poses a threat to public health worldwide. According to the World Health Organization (WHO)/World Organisation for Animal Health (OIE)/Food and Agriculture Organization of the United Nations (FAO) H5N1 Evolution Working Group, 10 distinct clades of these viruses (0–9) were initially designated in 2008 to characterize the phylodynamics of the hemagglutinin (HA) gene of the Gs/GD-like viruses circulating during 1996–2007 (*1*). On the basis of these nomenclature criteria, new second-, third-, and fourth-order clades have been identified within the previously defined clades in the phylogenetic analyses that were updated in 2009 and 2011 (*2*,*3*). Therefore, as HPAI A(H5N1) virus continues to undergo substantial evolution, extensive genetic divergence is expected to periodically accumulate to form novel monophyletic groups. To identify continued divergence of clade 2.3.4 other than the recognized subclades 2.3.4.1, 2.3.4.2, and 2.3.4.3, we characterized 7 HPAI A(H5N1) viruses isolated from poultry during 2009–2012 in China (*2*).

## The Study

As part of continuous national avian influenza virus surveillance, we performed a monthly collection of cloacal swabs from various poultry species (chicken, duck, goose, quail, and pigeon) at a wholesale live-bird market (LBM) in Yangzhou, Jiangsu Province, in eastern China. Birds offered for retail sale in the LBM were mainly from local farms in Jiangsu and the neighboring provinces in eastern China; some were transported from regions in southern or northern China. Virus isolation and identification were conducted as described (*4*). During December 2009–September 2012, avian influenza virus isolates belonging to 8 HA subtypes (H1, H3–H6, H9–H11) were identified; 7 of the isolates were subtype H5N1 (Table 1).

**Table 1:** Results of HI assays using Re-5 and k0602 antiserum for 7 avian influenza A(H5N1) viruses isolated in China, 2009–2012*

Isolate	Isolation date	Antibody titer, log_2_	
		Re-5	k0602
A/chicken/Jiangsu/WJ/2009(H5N1)	2009 Dec	4	8
A/chicken/Jiangsu/XZ/2010(H5N1)	2010 Mar	4	8
A/chicken/northern China/k0602/2010(H5N1)	2010 May	4	**10**
A/chicken/Shandong/k0603/2010(H5N1)	2010 Jun	4	10
A/chicken/eastern China/ZG56/2011(H5N1)	2011 Dec	4	5
A/chicken/eastern China/JX/2011(H5N1)	2011 Dec	3	7
A/chicken/eastern China/AH/2012(H5N1)	2012 Sep	4	1
Re-5 diagnostic antigen†	NA	**10**	4

*Re-5 and k0602 antiserum were generated by vaccinating specific-pathogen free chickens with the commercial Re-5 vaccine (Qingdao Yebio Bioengineering Co., Ltd, Qingdao, China) and the oil-emulsified inactivated A/chicken/northern China/k0602/2010(H5N1) vaccine, respectively. HI titers against the homologous antigen/virus are shown in **boldface**. HI, hemagglutination inhibition; NA, not applicable. †Qingdao Yebio Bioengineering Co., Ltd, China.

To characterize these 7 isolates, we sequenced the HA genes to determine clade distribution. In the reconstructed phylogenetic tree (Figure) using reference sequences retrieved from the GenBank database and partially recommended by WHO/OIE/FAO (*2*), the 7 isolates belonged to clade 2.3.4 (the “Fujian-like” sublineage), which has been prevalent in China since 2005 (*5*). However, none of the isolates could be further classified into previously identified subclades 2.3.4.1, 2.3.4.2, or 2.3.4.3. Six of the viruses closely resembled A/peregrine falcon/Hong Kong/810/2009, and the remaining virus was highly homologous with recent H5 viruses bearing various neuraminidase (NA) subtypes (N1, N2, N5, and N8).

**Figure:**
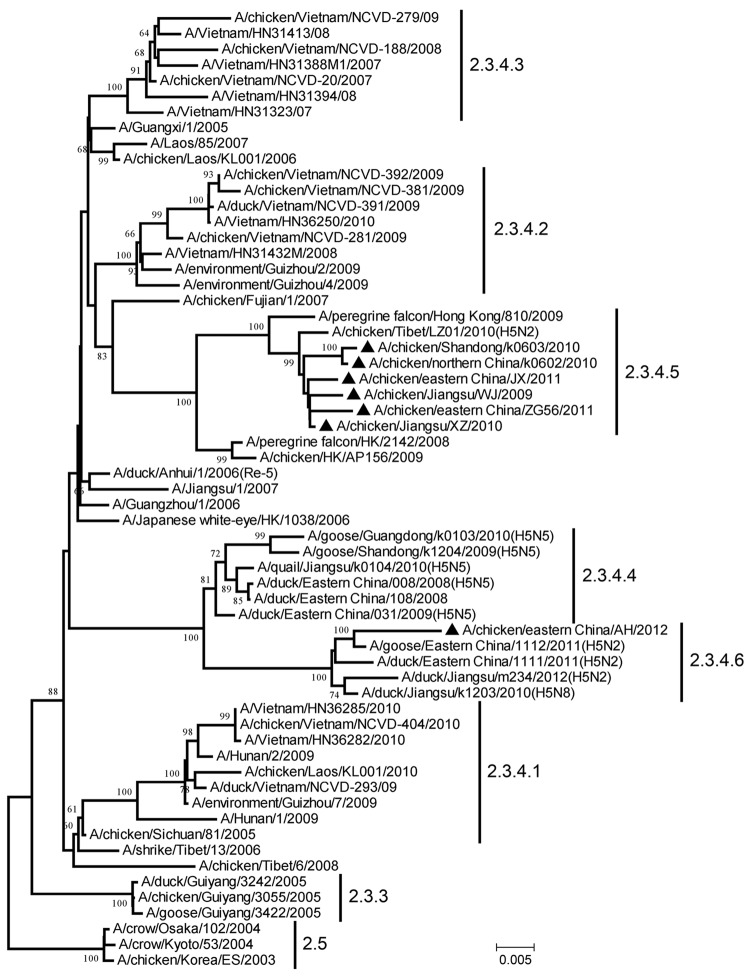
Phylogenetic tree of the hemagglutinin (HA) genes of the diverged avian influenza H5 subtype clade 2.3.4 variants from China and reference sequences retrieved from the GenBank database and partially recommended by the World Health Organization/World Organisation for Animal Health/Food and Agriculture Organization of the United Nations H5N1 Evolution Working Group The neighbor-joining tree was generated by using MEGA 5.1 software (www.megasoftware.net). Numbers above or below the branch nodes denote bootstrap values >60% with 1,000 replicates. Numbers on the right are existing (2.3.3, 2.3.4.1, 2.3.4.2, 2.3.4.3, 2.5) and proposed (2.3.4.4, 2.3.4.5, 2.3.4.6) virus subclades. Black triangles indicate the 7 variants identified in this study; GenBank accession numbers for their HA genes are KC631941–KC631946 and KC261450. Scale bar indicates nucleotide substitutions per site.

According to WHO/OIE/FAO guidelines (*1*–*3*), new clades (including subclades) were specified not only with a bootstrap value of >60 at the clade-defining node in which sequences monophyletically arose from a common ancestor but also with average between-clade and within-clade nucleotide divergences of >1.5% and <1.5%, respectively. Apart from subclades 2.3.4.1, 2.3.4.2, and 2.3.4.3, we found 3 additional monophyletic categories—the A/peregrine falcon/Hong Kong/810/2009-like viruses, the HPAI subtype H5N5–like reassortants, and the HPAI subtype H5N2/H5N8–like reassortants—that grouped clearly within the tree (Figure). The bootstrap values and average within-clade and between-clade distances for these 3 groups were 81, 1.0%, 4.2%; 100, 1.0%, 5.2%; and 100, 1.3%, 5.3%, respectively. 

Because of the compulsory vaccination practice against HPAI in China (*6*), we examined serologic cross-reactivity between the 7 subtype H5N1 isolates and the diagnostic antigen of the widely used inactivated reassortant H5N1/PR8 vaccine Re-5 (Table 1). Although Re-5 derived its HA and NA genes from a clade 2.3.4 representative virus A/duck/Anhui/1/2006, the hemagglutination inhibition (HI) titers of Re-5 antiserum against the 7 subtype H5N1 viruses were as much as 6–7 log_2_ lower than that against the homologous antigen. In contrast, the antiserum of A/chicken/northern China/k0602/2010 (k0602) showed limited reaction to Re-5 and A/chicken/eastern China/AH/2012. Moreover, antigenic variation also existed among the 6 A/peregrine falcon/Hong Kong/810/2009-like viruses, as highlighted by the HI assay using k0602 antiserum (Table 1).

To explore whether these antigenic variations can be translated into protection efficacy difference in vivo, we selected A/chicken/northern China/k0602/2010 (k0602) and A/chicken/Shandong/k0603/2010 (k0603) viruses to evaluate the bivalent inactivated reassortant H5N1/PR8 vaccine Re-4/Re-5 (the HA and NA genes of Re-4 are from a clade 7 virus A/chicken/Shanxi/2/2006). This vaccine has been extensively used to control the prevalence of clade 2.3.4 and clade 7 viruses in China since 2008 (*6*). In addition, a reassortant rk0602 virus, which carries the HA and NA genes of k0602 virus and the internal genes of PR8, was recovered by using reverse genetics and the inactivated rk0602 vaccine was applied to evaluate the homologous protection. Four-week-old specific-pathogen free chickens were vaccinated with Re-4/Re-5 or the rk0602 vaccine and readily developed specific antibodies against the component viruses by day 28 after vaccination (Table 2). The birds were then intranasally challenged with 10^6.0^ 50% egg infectious dose of k0602 or k0603 virus. During the 10-day observation period, the Re-4/Re-5 vaccinated birds displayed clinical signs including severe depression, ruffled feathers, huddling, decreased feed and water consumption, and diarrhea; moreover, only 14.3% (1/7 birds in the k0602 group) and 10% (1/10 birds in the k0603 group) of the challenged chickens survived, reflecting poor protection by the Re-4/Re-5 vaccine. In addition, shed virus was detected in tracheal and cloacal swabs from most of the tested chickens on 3 and 5 days postchallenge. By contrast, the rk0602-vaccinated chickens all survived the challenge, and no virus was recovered from tracheal and cloacal samples (Table 2).

**Table 2:** Efficacy of vaccines against highly pathogenic avian influenza virus A(H5N1) clade 2.3.4 variants in chickens, China*

Virus and vaccine type	HI titer ±SD, log_2_				Challenge test results, by swab type, no. positive birds/no. tested (mean titer ±SD)†					No. surviving birds/total no.
					3 dpc			5 dpc		
	Re-4	Re-5	k0602		Tracheal	Cloacal		Tracheal	Cloacal	
k0602										
Re-4/Re-5	8.10 ±0.97	6.20 ±0.31	2.25 ±0.45		6/6 (3.79 ±1.46)	4/6 (2.25 ±2.39)		2/3 (2.17 ±2.40)	2/3 (2.92 ±2.55)	1/7
rk0602	2.10 ±0.37	3.27 ±0.33	9.35 ±0.75		0/10	0/10		0/10	0/10	10/10
Control‡	ND	ND	ND		ND	ND		ND	ND	0/5
k0603										
Re-4/Re-5	7.70 ±0.26	5.70 ±0.29	1.93 ±1.21		8/8 (3.15 ±1.30)	8/8 (3.50 ±0.25)		3/3 (3.50 ±0.43)	3/3 (2.33 ±0.58)	1/10
rk0602	2.05 ±0.85	2.75 ±0.57	9.25 ±0.71		0/10	0/10		0/10	0/10	10/10
Control‡	ND	ND	ND		ND	ND		ND	ND	0/5

*Chickens were immunized with the Re4/Re5 or the inactivated rk0602 vaccine (the HA and NA genes of rk0602 were derived from subtype H5N1 k0602 virus; the internal genes were from PR8), and HI antibody titers were determined on day 28 postvaccination. HI, hemagglutination inhibition assay; dpc, days postchallenge; ND, not done. †Birds were challenged with 10^6.0^ 50% egg infectious dose (EID_50_) of k0602 or k0603 virus; virus titers are expressed as log_10_ EID_50_/0.1 mL. ‡Two groups of 5 mock-vaccinated chickens served as controls; all died within 3 dpc.

## Conclusions

The location of the 7 HPAI A(H5N1) virus variants in the HA gene tree (Figure) suggests that novel monophyletic subclades other than the previously identified 2.3.4.1, 2.3.4.2, and 2.3.4.3 subclades continue to emerge within clade 2.3.4. As a result of our findings, we suggest that these groups should be assigned new fourth-order clades of 2.3.4.4, 2.3.4.5, and 2.3.4.6 to reflect the wide divergence of clade 2.3.4 viruses.

In China, 1 of the 6 countries to which subtype H5N1 virus is endemic (*7*), multiple distinct clades (2.2, 2.5, 2.3.1, 2.3.2, 2.3.3, 2.3.4, 7, 8, and 9) were identified by surveillance during 2004–2009 (*5*). In particular, clades 2.3.2, 2.3.4, and 7 viruses have gained ecologic niches and have continued circulating by further evolving into new subclades (*2*). In addition, various NA subtypes of H5 viruses (H5N5, H5N8, and H5N2) bearing the genetic backbone of clade 2.3.4 A(H5N1) viruses have been detected in ducks, geese, quail, and chickens (*8*–*12*). These findings highlight the importance of periodic updates of the WHO/OIE/FAO classification of Asian A(H5N1) viruses by continuous surveillance to better understand the dynamic nature of the viral evolution.

Our findings have implications for the effectiveness of vaccination of chickens against HPAI A(H5N1) viruses. The results of cross-HI assays (Table 1) and vaccine efficacy experiments (Table 2) indicate antigenic drift in subtype H5N1 variants, as compared with the vaccine strain specifically designed to control the prevalent clade 2.3.4 virus infection in poultry. Although previous studies by Tian et al. (*13*) and Kumar et al. (*14*) proposed that vaccinated chickens with HI antibody titers of >4 log_2_ could be protected from virus challenge, our data demonstrate that vaccine efficacy is substantially influenced by antigenic matching between the vaccine strain and circulating viruses in preventing the replication and transmission of influenza virus, especially when the induced antibodies are of moderate titers.
